# ‘Forging healthy communities’: a service evaluation of a 12-week community-based exercise, nutrition, behaviour change and peer-support programme

**DOI:** 10.1186/s12889-025-22447-3

**Published:** 2025-04-09

**Authors:** Callum Leese, Blair H. Smith, Rosina Cross, Emma J. Cockcroft, Cassie Higgins

**Affiliations:** 1 Department of Population Health and Genomics, University of Dundee, Ninewells Hospital, James Arnott Drive, Dundee, DD2 4BF UK; 2 Department of Health and Community Sciences, University of Exeter Medical School, Exeter, UK

**Keywords:** Disease prevention, Exercise, Physical activity, Primary care, Health education, Health promotion

## Abstract

**Background:**

Physical inactivity is a leading cause of premature mortality and morbidity worldwide. Primary care settings provide an opportunity for effective lifestyle interventions, including physical activity (PA) promotion. This study aims to evaluate the impact of a rural community-based multi-component, 12-week exercise, nutrition, education and peer-support programme on participants health and wellbeing.

**Methods:**

This retrospective service evaluation included patients referred to the programme between January 2020 and December 2022 from primary care settings. Quantitative data (including body composition measures, mental wellbeing and patient activation) were collected at the entry and exit of the 12-week program. Participants also self-reported healthcare attendance in the 3 months prior to the baseline and post-intervention data-collection.

**Results:**

Of the 424 people who participated in the programme, 84.7% (*n* = 359) indicated that they had achieved their goals. Significant improvements in BMI, weight, blood pressure, wellbeing, patient activation, muscle mass, body-fat mass and reduced healthcare attendance over a 12-week intervention were identified by repeated measure ANOVA. Post-hoc tests with a Bonferroni correction found that younger participants were significantly more likely to decrease their BMI and increase their mental wellbeing (as measured by WEMWBS) over the course of the programme. Higher attendance at the programme was also associated with greater reductions in BMI and greater improvements in patient activation.

**Discussion:**

The findings support the effectiveness of multicomponent community-based exercise, nutrition, education and peer support interventions in improving health outcomes and reducing healthcare utilisation. Further research is needed to evaluate the long-term health outcomes of the education-exercise referral programme, across settings, and its potential to contribute to a sustainable healthcare system.

**Supplementary Information:**

The online version contains supplementary material available at 10.1186/s12889-025-22447-3.

## Background

Physical activity (PA) has well documented physical, psychological, and social benefits [1]. However physical inactivity is a leading risk factor for mortality and morbidity worldwide [2, 3], with 30% of European adults failing to meet recommended levels of PA [4]. Physical inactivity has implications beyond health, with physical inactivity related deaths contributing $13.7 billion in global productivity losses [5]. Increasing PA can also have significant environmental implications, such as a fall in vehicle use [6]. Lifestyle interventions via primary care have been shown to be effective at initiating behaviour change and reducing the risk of disease progression (for example cardiovascular disease and stroke) [7]. Several systematic reviews have shown that PA promotion delivered in primary care is effective at increasing PA levels [8–10], with an odds ratio of increasing PA at 12 months compared to no advice of up to 1.42 [11]. Furthermore, research has shown PA promotion within primary care to be a cost-effective intervention [12, 13] compared to usual care. Anokye and colleagues identified that brief advice for PA in primary care was a cost-effective intervention.

Despite evidence of the clinical and cost-effectiveness of primary care-based PA interventions [14, 15], evidence shows primary care professionals are not delivering PA promotion [16, 17]. This implementation gap has been explored through a number of systematic reviews, which have highlighted multiple barriers to PA promotion in primary care, including a lack of time, resources and knowledge [18]. Exercise referral schemes (ERSs) have the potential to address many of these challenges, supporting primary care to deliver PA promotion. However, the evidence base for ERSs is lacking, with only weak evidence supporting their effectiveness at improving some markers of health and well-being [19–21]. Cost-effectiveness of ERSs in primary care were evaluated by Campbell and colleagues [22], finding that despite small improvements in PA levels they are not cost-effective. Several limitations within the current literature exist, including lack of methodological reporting and consistency in the collection and reporting of outcomes. Although most ERSs focus solely on PA [19, 23], recent research has shown that integrating PA with nutrition, is more effective at helping participants lose weight, than delivering physical activity interventions on their own [24]. Cardiac rehabilitation is an example of an integrated intervention (combining exercise with education sessions and peer support [25]), and has been shown to reduce mortality, hospital admission and improve both psychological wellbeing and quality of life [25]. The peer support involved within ERSs has been highlighted as an important facilitator of engagement in a previous systematic review by Eynon and colleagues in 2019 [26]. This aligns with previous research showing that community connectedness is associated with improved health and wellbeing [27] and that increased community connectedness is considered a key benefit associated with increased physical activity [28].

Despite this, little research exists evaluating an integrated approach for primary care and community based ERSs. Given the failures of the implementation of PA promotion in primary care this study is a service evaluation of an established ERS integrating nutrition, education, behaviour modification and peer support alongside physical activity among participants referred from primary care, aiming to evaluate its the impact on participants’ health and wellbeing.

## Methods

### Participants, setting and design

The Lorn and Oban Healthy Options (LOHO) is a community-based charity located in the west of Scotland. It delivers a 12-week integrated approach to an ERS (delivered by a health and wellbeing practitioners), designed to improve the health and wellbeing of participants. Referral is made by: a medical professional; a housing officer; an employee of defined local third sector organisations; or by self-referral. Individuals can self-refer by contacting the charity through their website or by telephone. Participants must be aged 16 years or above and have physical, psychological or social needs that could be met by participation in the programme and not be acutely medically unwell. There are no other exclusion criteria for this service. Need is either self-identified or identified at point of referral in discussion with a healthcare professional or one of the other people listed above. All individuals were contacted following referral by programme staff to vet for appropriateness and to obtain informed consent to participate the programme. This study presents an outcome evaluation of all patients referred to the LOHO programme between 1 January 2020 and 31 December 2022.

### Intervention

**Fig. 1:**
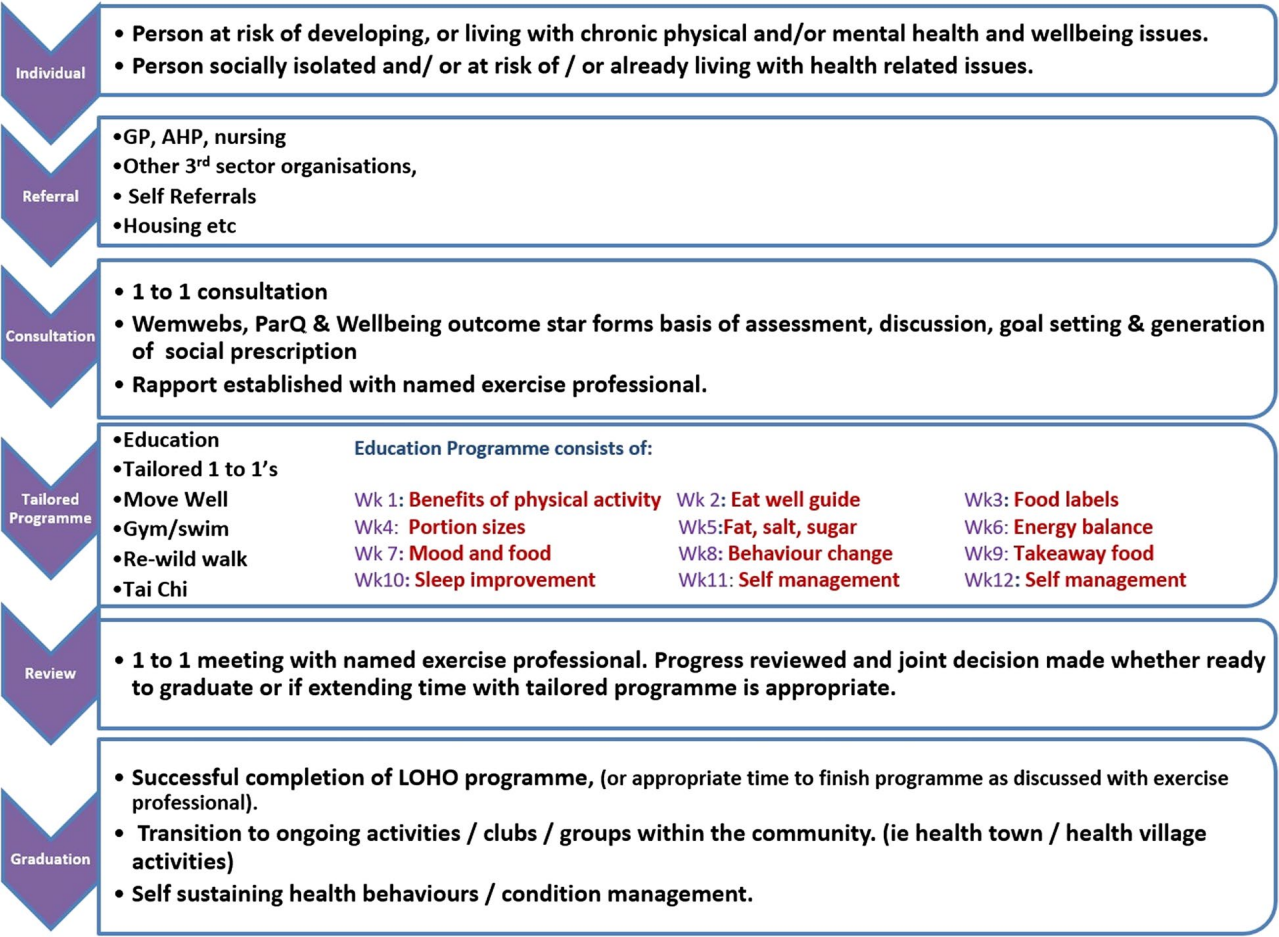
Overview of the LOHO model [image reproduced with the permission of the Development Manager, © 2022 by Lorn and Oban Healthy Options, Ltd., licensed under CC BY-NC-ND 4.0]. The programme, initially established in 2012, is a 12-week structured integrated ERS, including nutrition, education, behaviour modification and peer support. Individuals can be referred a medical professional; a housing officer; an employee of defined local third sector organisations; or by self-referral. The figure shows the participant pathway, with an initial consultation and progress review book-ending a 12-week tailored programme incorporating 3 sessions per week (2 physical activity based and one educational). Abbreviations: GP: general practitioner; AHP: allied health professional; WEMWBs: Warwick and Edinburgh mental wellbeing score; PARQ: physical activity readiness questionnaire; LOHO:; Lorn and Oban Healthy Options

The Lorn and Oban Health Options programme, initially established in 2012, is a 12-week structured integrated ERS, including nutrition, education, behaviour modification and peer support. This innovative model has been developed as part of an iterative process since its inception in 2012, allowing for the implementation of new research and best practice whilst tailored to the specific needs of the community. The programme includes an initial 1:1 consultation (with a health and wellbeing practitioner) to collect baseline data (see Fig. 1) and to tailor the core programme to meet individual needs. The programme includes twice-weekly group-based exercise classes (including strength gym-based classes, Tai Chi, walking and swimming) and once-weekly educational workshops that integrate behaviour change techniques. The behaviour modification strategies are based on the motivational, action, prompts (MAP) model for behaviour change produced by NHS Education Scotland [29]. Individuals are educated in the MAP model and encouraged to co-produce smart, measurable, achievable, relevant and time-bound (SMART) goals alongside exercise specialists. Given the three sessions per week and the 1:1 reviews at the beginning and end of the programme, the total number of attendances is 38. It is delivered by a team of exercise specialists (n = 5) trained in accordance with Chartered Institute for the Management of Sport and Physical Activity (CIMSPA) guidance to work with people with chronic diseases. CIMSPA is the professional body for the sport, fitness and physical activity sector in the UK. Participants are encouraged to attend as many of the three weekly events as they can, but there is no mandated or minimum attendance. Care is also taken to account for individuals’ health, economic and social circumstances during the initial 1:1 consultation. The 12-week educational programme is highlighted in Fig. 1. During the intervention period, classes were delivered using a combination of face-to-face and online formats seeking to address equality of access. For individuals who cannot attend face-to-face sessions (for example due to health, geography or caring responsibilities), home-based exercise programmes are provided. Programme exit/completion is determined collaboratively between participant and the staff member (health and wellbeing practitioner) at the 12 week 1:1 review (or earlier if requested by the participant). Programme completion is defined as participants having completed the 12-week programme or having met their intended goals and intentions, pre-determined at the 1:1 review with an exercise professional upon enrolment. Participants are able to attend for more than 12 weeks if, after review with an exercise professional, it is felt there was potential for ongoing benefit. This flexibility was particularly valuable during the COVID-19 pandemic, with attendance at the ERS often interrupted. Lorn and Oban Healthy Options run other exercise programmes for individuals needing less support, with participants encouraged to attend these following the completion of the 12-week ERS. An overview of the healthy options model is shown in Fig. 1, with further details also provided in Supplementary File 1 (template for intervention description and replication (TIDieR) checklist and guide).

### Outcome measures

The initial referral provided information on sex, age and reason for referral. All other measures were performed by a health and wellbeing practitioner at the initial and the final consultations, although completion of dataset was not mandated. Basic information (height, weight, body mass index (BMI), blood pressure and resting heart rate) was measured. Body composition was measured using the Tanita system of bioelectrical impedance analysis (BIA) (model TBF-400), which employs multiple frequencies to measure body composition. The Tanita TBF-400 reports fat-free mass (FFM), which includes lean soft tissue (including skeletal muscle, smooth muscle, organs and connective tissue) and bone minerals. Tanita machines are unable to explicitly separate skeletal muscle for other components of lean soft tissue. Fat-free mass and body fat mass were measured in kilograms (kg), and these values were converted to percentage of body weight. The Tanita machine assigns a score for visceral fat level (VFL) ranging from 1 (the lowest) to 59. All measurements were taken following a pre-specified protocol and using calibrated instruments.

Two standardised questionnaire instruments were used by programme staff (trained exercise professionals) to collect data during both the initial and final consultations. First, the Patient Activation Measure – short form (PAM-SF-SF) [30], is a validated 13-item questionnaire designed to assess a participant’s ability and willingness to take independent actions to manage their health and wellbeing. The PAM-SF provides an individual ‘activation’ score on a 0–100-point scale, and higher scores indicate greater activation. Secondly, the Warwick-Edinburgh Mental Wellbeing Scale (WEMWBS; [31]), a validated 14-item instrument intended to assess mental wellbeing in the general population. Each item has 5 response categories, which are summed to compute a single total score. Total scores range from 14 to 70, and higher total scores are associated with greater positive mental wellbeing.

A service-designed survey instrument was used to collect additional data during the initial and final consultations. This included patients’ perceptions of their general health and community connectedness (0–5 scale, with higher scores reflecting more positive perceptions), the number of healthcare services involved with the patient, and the number health service attendances during the preceding 3 months.

For each patient referred to LOHO, all data were routinely collected by the service. Data were stored securely using Elemental (Access Group, Loughborough), in accordance with NHS Highland data storage and protection policy. On securing the appropriate ethics approvals, this data, spanning the period 1 January 2020 to 31 December 2022, was anonymised, cleaned and coded before being transferred to the University of Dundee for the purpose of conducting the present evaluation.

### Statistical analysis

Data were analysed using SPSS v28. The descriptive statistics concerning sex and referral reason were reported as frequency and percentage of the cohort. The descriptive statistics concerning age and number of referral reasons per person were reported as mean and standard deviation (SD). Post-intervention outcome (needs met, referred to another service, etc.) were reported as frequency and percentage of the cohort.

The proportion of the cohort that achieved clinically significant change on body composition and clinical outcome measures was reported as frequency and percentage of the cohort. Thresholds for clinically significant change, where available, were obtained from the existing literature: (1) a 5% reduction in BMI [32]; (2) in the absence of available data for muscle mass, body fat mass and visceral fat, the threshold for BMI (i.e. a 5% reduction) was used; (3) a reduction of ≥ 10 mmHg in systolic BP or a reduction of ≥ 5 mmHg in diastolic BP [33]; (4) [33]; a ≥ 3-point improvement on WEMWBS [34]; and (5) a ≥ 4-point improvement on PAM-SF [35–37].

Pre- and post-intervention comparisons were conducted for BMI, body composition, blood pressure, resting heart rate, WEMWBS score, PAM-SF score and number of social and physical activities per week. The statistical significance was determined using repeated measures analysis of variance (ANOVA) tests, with a Greenhouse–Geisser correction to adjust for violations of sphericity. The findings were reported as pre-intervention mean and SD, post-intervention mean and SD, statistical significance between pre- and post-intervention values (*p*-value) and effect size estimate (partial eta squared $\left({\eta }_{p}^{2}\right)$). Effect size estimates were reported to provide an estimate of clinical significance, and interpretation of ${\eta }_{p}^{2}$ is as follows: small effect (0.01 to 0.05); medium effect (0.06 to 0.13); and large effect (≥ 0.14) [38].

*Post-hoc* analyses with a Bonferroni correction were conducted for the same outcome measures, examining associations with the following interactions: (1) time by age group; (2) time by total number of attendances at the intervention; and (3) time by participation / no participation in activities at baseline. These variables were deemed to be of particular clinical interest and were identified a priori. The findings were reported as pre-post intervention mean differences, statistical significance (*p*-value) and effect size estimate $\left({\eta }_{p}^{2}\right)$.

The nonparametric Wilcoxon signed ranks test was performed to examine pre-post intervention change in perceptions of community connectedness, and the finding was reported using the Z statistic. Unhappiness with a post-intervention decline in community connectedness (in those that reported a decline) was investigated, and the findings were reported as frequency and percentage. General health and BMI was investigated in those that reported a post-intervention increase in community connectedness, and the findings were reported as frequency and percentage.

Pre- and post-intervention comparisons of healthcare utilisation were computed using repeated measures ANOVA with a Greenhouse–Geisser correction. The outcome measures were: (1) number of healthcare services involved; (2) number of GP appointments; (3) number of emergency department attendances; and (4) number of inpatient admissions. The findings were reported as pre-intervention mean and SD, post-intervention mean and SD, statistical significance between pre- and post-intervention values (*p*-value) and effect size estimate $\left({\eta }_{p}^{2}\right)$

## Results

During the observation period, 627 clients were referred. Of those referred, 424 participants were enrolled in the programme, and included in this analysis. Reasons for failure to enrol included: declined input (*n* = 78), not contactable (*n* = 52), no longer required the service (*n* = 27), sign-posted to more appropriate service (*n* = 22), failed to attend (*n* = 20) and other reasons (*n* = 4). Table 1 shows the demographic and treatment characteristics Table 1.

**Table 1:** Sociodemographic and treatment characteristics among Healthy Options participants (*N* = 424)

Characteristic	Frequency (%) or mean ± SD
Sex (n, %)	
Female	241 (56.8)
Male	183 (43.2)
Age (mean ± SD)	58 ± 17 years
Total number of programme attendances (mean ± SD) *	14.8 ± 14.1 attendances
Reason for referral (n, %) **	
Management of chronic medical conditions	205 (48.3%)
Management of physical wellbeing	77 (18.2%)
Management of mental wellbeing	154 (36.3%)
Management of pain problems	84 (19.8%)
Weight management and dietary guidance	209 (49.3%)
Rehabilitation medicine	155 (36.6%)
Not known *	65 (15.3%)
Number of referral reasons per person (mean, SD)	2.24 ± 1.06

^*^This figure refers to the number of attendances at individual and group sessions but, since the observation period coincided with COVID-19 lockdowns, many of the aspects of the intervention were home-based, and these sessions are not included in this figure. Additionally, home-based sessions are routinely delivered to accommodate people’s other commitments and circumstances. In consequence, this figure does not reflect engagement with the intervention but, rather, attendance at sessions in the presence of others. ** In most cases the referral reason was recorded as ‘social prescribing’; however, this indicates both the referral route (i.e. referred by social prescribers) and the method of deliver of healthy options rather than the referral reason

Fifty-seven percent of participants were female. The mean age of participants was 58, with an age range of 16–96 years. The mean number of programme attendances was 14.8 (38 possible across the twelve weeks, however given the ability to remain within the programme for more than 12 weeks outliers exist). Participants reported an average of 2.24 reasons for referral, with chronic medical condition management (48.3%) and dietary guidance (49.3%) being most common.

Table 2 shows pre- and post-intervention change of body composition and clinical characteristics.

**Table 2:** Pre- and post-intervention change of body composition and clinical characteristics for participants of the Healthy Options programme (using repeated measures ANOVA with a Greenhouse–Geisser correction)

**Clinical characteristic**	**Pre-intervention mean (SD)**	**Post-intervention mean (SD)**	***p-value***	$\left({{\varvec{\eta}}}_{{\varvec{p}}}^{2}\right)$
Body Mass Index (BMI; kg/m2) [n = 227], *	32.50 (8.22)	32.03 (7.29)	0.034	0.020
Percentage of fat-free mass [n = 206]	61.94 (10.35)	63.18 (9.85)	< 0.001	0.089
Percentage body fat mass [n-212]	34.61 (9.94)	33.70 (10.07)	< 0.001	0.064
Visceral fat level [n = 211]	13.17 (5.29)	13.01 (5.36)	0.008	0.033
Systolic blood pressure (mmHg) [n = 206]	141.82 (17.60)	138.53 (16.32)	0.006	0.036
Diastolic blood pressure (mmHg) [n = 206]	82.95 (10.30)	81.74 (9.47)	0.068	0.016
Resting heart rate (bpm) [n = 202]	73.77 (12.01)	71.79 (11.22)	0.005	0.039
WEMWBS score [n = 336]	47.44 (10.16)	53.65 (9.64)	< 0.001	0.326
PAM-SF score [n = 74)	60.74 (12.62)	68.03 (15.59)	< 0.001	0.169
Number of social and physical activities per week [n = 313]	1.77 (2.30)	2.97 (2.62)	< 0.001	0.238

Datasets were incomplete for three main reasons; non-completion secondary to it not being mandated, COVID-19 impacting data collection, and an instrument (PAM-SF) being phased out over the course of the evaluation period. This is addressed more thoroughly in the results

^*^Two participants were removed from this analysis due to being underweight at baseline and aiming to gain weight as a function of the intervention. Both participants gained weight during the intervention

The intervention yielded significant improvements in various clinical metrics, as demonstrated by repeated measures ANOVA. Participants experienced reductions in BMI (p = 0.034), visceral fat levels (p = 0.008), and resting heart rate (p = 0.005), alongside increases in fat-free muscle mass (p < 0.001). Mental well-being and patient activation improved, with WEMWBS and PAM-SF scores increasing significantly (p < 0.001). Enhanced engagement in weekly social and physical activities (*p* < 0.001) was also observed.

Table 3 shows post-intervention outcome and clinically significant changes in treatment outcomes at intervention completion.

**Table 3:** Post-intervention outcome and clinically significant change in treatment outcomes at completion of the Healthy Options programme

Outcome	Frequency (%)
Post-intervention outcome*	
Needs met	359 (84.7)
Referred to other service	53 (12.5)
Engaged in physical ± social activity elsewhere	4 (0.9)
Not specified	8 (1.9)
Clinically significant change achieved on treatment outcome measures	
Decreased body mass index	17 (7.4)
Increased percentage of fat-free mass	29 (14.1)
Decreased percentage of body fat mass	56 (26.3)
Decreased visceral fat level	59 (28.0)
Decreased blood pressure	113 (54.9)
Decreased resting heart rate	63 (31.2)
Increased mental wellbeing	27 (67.6)
Increased patient activation	40 (54.1)

^*^4 outcomes were identified at exit from the programme; needs met (successful attainment of goals identified at initial 1:1 consultation), referred to other service (in order to address unmet needs or ongoing support), engaged in physical ± social activity elsewhere (acting as an alternative solution to help the patient attain their goals) and not specified

Thresholds for clinically significant change, where available, were obtained from the existing literature: (1) a 5% reduction in BMI (2) in the absence of available data for muscle mass, body fat mass and visceral fat, the threshold for BMI (i.e. a 5% reduction) was used; (3) a reduction of ≥ 10mmHg in systolic BP or a reduction of ≥ 5mmHg in diastolic BP; (4); a ≥ 3-point improvement on WEMWBS; and (5) a ≥ 4-point improvement on PAM-SF

Eighty-five percent (*n* = 359) completed the programme (defined as ‘having completed the 12-week programme or having met their intended goals and intentions’). Fifty-three (12.5%) were referred to another service, four (0.9%) reported not completing due to engaging in physical and/or social activities elsewhere, with the final eight (1.9%) participants having no specified reason for failure to complete.

During the 12-week intervention, clinically significant improvements were observed across multiple metrics: 54.9% (*n* = 113) achieved reduced blood pressure, and 31.2% (*n* = 63) achieved a lowered resting heart rate. Substantial changes in body composition were noted, including a decrease in visceral fat in 28.0% (*n* = 59) of the cohort. Mental wellbeing and patient activation scores (as measured by PAM-SF) also showed marked improvement (with clinically significant change having been achieved in 67.6% (*n* = 27) and 54.1% (*n* = 40) of the cohort, respectively).

Post-hoc analyses with a Bonferroni correction were conducted to further investigate the findings reported in Table 3. These findings were stratified by sex, age group, number of attendances and baseline activity, which are variables that were considered to be of interest to service providers. There was no effect of time x gender on any of the outcome measures. The remainder of the findings are shown in Table 4. Table 4 shows that post-hoc analyses indicated differential program associations on the outcome measures. Younger adults showed greater BMI reductions compared to older adults (*p* = 0.010). Participants that attended on more than 10 occasions were associated with a greater reduction in BMI (*p* = 0.022), a greater increase in patient activation (*p* = 0. 040) and a greater number of social and physical activities (*p* = < 0.001)(when compared to those who attended on 10 or less occasions). Participants who were not engaged in any activities at baseline had significantly greater reductions in resting heart rate (*p* = 0.011) and greater increases in number of social and physical activities undertaken (*p* < 0.001).

**Table 4:** Mean differences on *post-hoc* repeated measures analysis of variance with Bonferroni correction for time by age group, time by total number of attendances at intervention and time by engaging with any social or physical activities per week at baseline for participants of the Healthy Options programme

**Variable**	Time by age group *					Time by total attendances **				**Time by any baseline activities**			
	**Young adults**	**Middle adults**	**Older adults**	***p*-value**	${\eta }_{p}^{2}$	**Up to 10**	**11 or more**	***p*-value**	${\eta }_{p}^{2}$	**No**	**Yes**	***p*-value**	${\eta }_{p}^{2}$
BMI (kg/m2)	−2.660	−0.372	−0.166	**0.010**	**0.040**	−1.175	−0.133	**0.026**	**0.022**	−0.688	−0.307	0.394	0.003
FFM (%)	+ 0.412	+ 1.480	+ 1.123	0.534	0.036	+ 1.655	+ 1.043	0.305	0.005	+ 1.123	+ 1.122	0.861	< 0.001
BFM (%)	−0.7005	−0.840	1.021	0.906	0.001	−0.907	−0.903	0.993	< 0.001	−0.795	−1.153	0.415	0.003
VFL	+ 0.055	−0.173	−0.179	0.544	0.006	−0.164	−0.154	0.906	< 0.001	−0.221	−0.142	0.469	0.003
SBP (mmHg)	−4.200	−3.139	−3.311	0.975	< 0.001	−3.890	−3.034	0.743	0.0101	−4.582	−3.011	0.518	0.002
DBP (mmHg)	−3.667	−2.040	+ 0.145	0.159	0.018	−0.163	−1.650	0.298	0.005	−0.280	−1.944	0.220	0.007
RHR (bpm)	−2.812	−1.949	−1.862	0.939	0.001	−3.370	−1.414	0.197	0.008	−4.234	−0.622	**0.011**	**0.032**
WEMWBS	+ 10.297	+ 6.149	+ 5.167	**0.008**	**0.029**	+ 5.800	+ 5.524	0.466	0.002	+ 5.483	+ 6.541	0.293	0.003
PAM	−6.167	+ 8.904	+ 8.092	0.104	0.062	+ 2.332	+ 10.302	**0.040**	**0.057**	+ 6.926	+ 7.610	0.858	0.001
Activities	+ 0.853	+ 1.290	+ 1.161	0.539	0.004	+ 0.671	+ 1.584	** < 0.001**	**0.043**	+ 1.854	+ 0.791	** < 0.001**	**0.057**

*BMI* Body Mass Index, *FFM* fat-free mass, *BFM* body fat mass, *VFL* visceral fat level, *SBP* systolic blood pressure, *DBP* diastolic blood pressure, *RHR* resting heart rate; *Gen Health* general health, *WEMWBS* Warwick-Edinburgh Mental Wellbeing Scale, *PAM* Patient Activation Measure, Activities = number of social and physical activities per week. *p*-values shown in bold are statistically significant. *Young adults: 16–35 years; middle adults: 36–65 years; older adults: > 65 years. **Total number of attendances was split around the median

A Wilcoxon signed-rank test was performed to examine pre-post intervention change in perceptions of community connectedness [*n* = 313]. By the end of the intervention, the median value had decreased significantly (z = −6.21; *p* < 0.001) from 2.0 (IQR = 2.0) to 1.0 (IQR = 1.0), with 35 (11%) reporting a higher post-treatment score, 105 (34%) reporting a lower post-treatment score and 173 (55%) reporting no change. Of the 105 participants that reported a post-intervention decline in community connectedness, only 6 (5.7%) were unhappy with this, and only 1 (1.0%) desired change. However, a significantly (χ^2^(2) = 7.73; *p* = 0.021) higher proportion of those that reported increased community connectedness (20%, *n* = 7) reported a post-intervention improvement in general health compared with those that reported no change (9%, *n* = 15) or a decrease in community connectedness (5%, *n* = 5). A significantly (χ^2^(2) = 7.58; *p* = 0.023) higher proportion of those that reported increased community connectedness (20%, *n* = 4) also experienced a clinically significant reduction in BMI compared with those that reported no change (4%, *n* = 4) or a decrease in community connectedness (10%, *n* = 8).

Table 5 shows pre- and post-intervention comparisons of number of health service attendances.

**Table 5:** Pre- and post-intervention comparisons of healthcare attendances for Healthy Options programme participants (using repeated measures ANOVA with a Greenhouse–Geisser correction)

**Clinical characteristic**	**Pre-intervention mean (SD)**	**Post-intervention mean (SD)**	***p-value***	${\eta }_{p}^{2}$
Number of healthcare services involved [number of participants reporting outcome = 313]	1.04 (0.93)	0.85 (0.80)	< 0.001	0.041
Number of GP appointments [number of participants reporting outcome = 174]	2.05 (2.44)	0.79 (1.56)	< 0.001	0.196
Number of emergency department attendances [number of participants reporting outcome = 174]	0.17 (0.47)	0.05 (0.29)	< 0.001	0.068

The self-reported healthcare attendance was significantly decreased post-intervention both in terms of the number of health services involved and the number of contacts with GPs, emergency departments and inpatient facilities.

## Discussion

### Summary

Despite small effect size, the retrospective service evaluation design and the impact of COVID-19 during the study period, the results of our study show a number of positive trends. They demonstrate that, among participants referred to the LOHO programme, there was an improvement in markers of health and well-being, including BMI, fat-free mass, body-fat mass, weight, blood pressure, resting heart rate and mental wellbeing. Following the intervention, a decrease in healthcare-seeking behaviour was observed, which correlates with improvements in patient-activation (as measured by the PAM-SF score).

### Comparison to existing literature

The sample demographic data are similar to those published from the Welsh National ERS [39], with similar age (mean age and SD) and female sex predominance. The Welsh Exercise Referral Scheme is a national 12-week initiative to improve PA levels in people with long-term health condition through structured exercise program provision. Of the 627 referred to the ERS, there was a 68% (n = 424) enrolment. That 68% were enrolled may have been impacted by organisational administration, with all referrals first vetted for appropriateness (with participant contact as appropriate) prior to invitation to the ERS (with redirecting elsewhere if more appropriate). The rate of completion in those enrolled in this study (84.7%) is higher than previously documented [39, 40], and may reflect that ‘completion’ was determined when a participants ‘needs’ (identified through 1:1 meetings with health and wellbeing professionals during the enrolment process) were met, as opposed to having attended all parts of the 12 week programme. The favourable completion rate compared with the Welsh scheme (43.8%) for example [39], may also in part be explained by the person-centred flexible delivery of LOHO, whilst the Welsh scheme was a research-focused randomised controlled trial (RCT).

Effectiveness of integrated lifestyle interventions (including physical activity, diet and behavioural change elements) have good evidence across a range of different conditions, including diabetes [41, 42], weight management [43], severe mental illness [44] and cardiovascular disease [45, 46]. This has resulted in lifestyle interventions becoming the standard for effective strategies to prevent and manage a range of conditions, notably cardiac rehabilitation programmes and both diabetes remission and prevention programmes. Cardiac rehabilitation programmes (combining exercise with education sessions and peer support) have been shown to reduce mortality and hospital admission, and improve psychological wellbeing and quality of life [25]. However, despite the apparent uniformity of many of these lifestyle interventions, they are often limited to specific population groups. For example cardiac rehabilitation is available only to those who have recently had a cardiac event. There is limited evidence for population-based interventions delivered simultaneously to individuals with a range of conditions, as done within the LOHO model. This is important in the context of growing multi-morbidity and limited availability, with the potential to facilitate widening access. The LOHO model offers a promising example of the potential of integrated ERSs for a much wider population group, but further research is required to account for the confounding factors (e.g. competing health interventions, self-reporting bias, COVID-19) within this service evaluation, and to compare the effect of a LOHO-style wide population integrated ERS with more traditional condition-specific ERSs.

Our results suggest a differing association with age on specific outcome measures. A narrative review in 2013, by Bouchard and colleagues [47], highlighted a trend towards improved health outcomes for older people following lifestyle interventions (with particular reference to reduction in body weight, blood pressure and type 2 diabetes risk). A number of possible explanations for these differences in outcomes by age may exist, including: (a) differences in baseline risk, with higher risk of long-term conditions and multimorbidity in older populations; (b) increased adherence and motivation in retired adults who may have fewer competing commitments than younger adults do; (c) physiological differences, for example hormonal and metabolic rate changes [48, 49] with age; and (d) opportunities, with recent research showing that older adults are comparatively more likely to be referred to exercise referral schemes than younger adults are [50]. Interestingly, the findings from Bouchard and colleagues stands in contrast to our work, where younger adults were found to have a greater BMI reduction than middle-aged and older adults. This may have been in part influenced by reasons for referral, with our research having wider inclusion criteria (including a number of referrals for sarcopenia in the elderly) compared to the review (which focussed mainly on obesity). In addition, improvements in mental wellbeing (as measured by WEMWBS) were greater in young adults. This may be explained by significantly more younger adults being referred to the service for mental wellbeing than middle-aged or older adults, or it could be an independent effect. The implications of this could be important, opening the potential for targeted and precision-based approaches that account for factors such as age and reason for referral. This aligns with the principles of personalised care [51], highlighted as of key pillar of the NHS England ‘Long Term Plan’ [52]. However, given the limitations of this service evaluation, further research is required to explore the role and effectiveness of targeted approaches.

One of the aims of the education component is to improve patient activation and self-empowerment regarding health [53]. We found a significant increase in mean PAM-SF score by the end of the 12-week period. An increase in number of social and physical activities attended per week was reported following the intervention, supporting the findings of improved patient activation and self-empowerment. Additionally, our analysis highlighted that greater numbers of attendances at the service resulted in greater improvement in PAM-SF scores and a greater number of social and physical activities undertaken. Existing evidence around patient activation and ERS is limited, but these promising findings suggest a role for patient activation in encouraging health-related behavioural change.

In contrast to the findings showing an increase in the number of social and physical activities, respondents reported that their perceptions of community connectedness had decreased by the end of the 12-week period. This perceived contradiction warrants exploration through further research, particularly given the substantial negative impact of loneliness on all-cause mortality [54]. Social engagement is one of the key facilitators of engagement in lifestyle interventions [55], with standard practice often looking to deliver lifestyle interventions in group context [25, 56]. We propose two possible explanations for this contradiction between increased number of social activities and perceived decreasing community connectedness. First, the evaluation was performed between 2020 and 2022 amid the global COVID-19 pandemic and the consequential lockdowns and enforced isolation. As a result, participants’ activities outside of the LOHO programme may have decreased, with travel restrictions also impacting time spent in diasporic communities (for example family). Secondly, as individuals became increasingly aware of the importance of social connectedness through the education programme they may have come to perceive their social connectedness differently, a theory proposed in health-belief model [57].

Acknowledging the low numbers, our findings report a decrease in GP, emergency department and healthcare attendance. These findings are noteworthy but may be in part due to the COVID-19 pandemic. During the first 12 months of the COVID-19 pandemic, much elective healthcare was paused and healthcare attendance in the UK, particularly in primary care, decreased [58]. Consequently, further research is required to assess the impact of the LOHO programme on healthcare attendance, which could be combined with an assessment the long-term impact and an economic analysis of the programme. However general practice services in the UK are currently in crisis, a combination of increased numbers of patients, complexity of consultations, relative fall in funding, staffing shortages and a recent pandemic [59–61]. Consequently, enabling people to manage their own health is vital, particularly given the strong association between improved health outcomes and decreased healthcare costs with increasing patient activation [53].

### Strengths and weaknesses

Despite the opportunistic nature of the study, a range of outcome measures was assessed in all individuals who participated in the intervention during the observation period included. This was an opportunistic pragmatic study and not a randomised-controlled trial. Because of this, rigorous scientific input around the planning and design and conduct phases was not practical. Despite this, the outcomes of this service evaluation do offer an insight into real-world application and effectiveness of a community based and funded ERS, and findings can inform future, more formal research.

Secondly, there was a lack of follow-up of ongoing health outcomes beyond completion of the programme. This limits the ability to discuss sustained behaviour change outcomes. This was a pragmatic decision given the financial constraints of the charity, and future research is required to assess the long-term impact of this programme.

The low response rate to the PAM-SF instrument is largely explained by the measure being phased out during the observation period. It was replaced, after the intervention period, by a physical literacy assessment tool as outlined in the Public Health Scotland physical activity referral standards, published in the same year [62].

Furthermore, the study period was impacted by the COVID-19 pandemic and subsequent lockdowns. The programme delivery continued with adaptions, including online delivery for 12 months (March 2020-March 2021). The impact of the COVID-19 pandemic on health-outcomes is being increasingly documented [63]. COVID-19 itself resulted in increased mortality and morbidity, whilst indirect effects on health included pressures on the healthcare service (with 7.5 million fewer referrals to consultant-led elective care in the first 15 months), significant reductions in mental health, lost learning from those in education and a decrease in both employment and economic activity [63]. With relation to this study the COVID-19 pandemic is likely to have significantly impacted findings, with results needing to be interpreted in light of this. This may be particularly relevant when considering the following outcome measures: community connectedness, attendance at healthcare services and engagement with social activities. As discussed previously, the pandemic and lockdowns resulted in a fundamental shift in social interactions and community (with many social engagements being moved online or cancelled) as well as healthcare delivery (elective work being cancelled and digitalisation where able of healthcare delivery). Given the profound impact on all aspects of society the pandemic is likely to have influence results (in both predicted and unpredicted ways). The adaptation of the programme to transition online impacted the ability to collect certain data (e.g. weight, visceral fat), but its inherently flexibility allowed for a person-centred approach with tailoring to individual needs at all levels (input, outcome, in-person or online). As previously stated, the dataset was incomplete, in part, due to the COVID-19 pandemic but also because completion of self-administered questionnaires was not compulsory.

## Conclusion

The LOHO ERS offers a novel insight into the potential of integrated exercise referral schemes (including nutrition, education, behaviour modification and peer support), finding improved markers for health and wellbeing and decreased help-seeking behaviour. Further research is required to build on this service evaluation, addressing the limitations of our study design, and assessing the long-term impact of the programme. However, given the current political agenda for preventive health and the possibility of cost-saving through decreasing healthcare attendance, these findings are of potentially important value.

## Supplementary Information

Supplementary Material 1.

## Data Availability

No datasets were generated or analysed during the current study.

## Abbreviations

WEMWBS: Warwick and Edinburgh Mental Wellbeing Score
LOHO: Lorn and Oban Healthy Option
PA: Physical activity
BMI: Body mass index
ERS: Exercise referral scheme
BIA: Bioelectrical impedance analysis
VFL: Visceral fat level
PAM-SF: Patient activation measure
RCT: Randomised controlled trial
