# Supplementary material for: ‘Forging healthy communities’: a service evaluation of a 12-week community-based exercise, nutrition, behaviour change and peer-support programme
Source: BMC Public Health. 2025 Apr 9;25:1326. doi: 10.1186/s12889-025-22447-3 (PMC11980141; doi:10.1186/s12889-025-22447-3)
Supplement: Supplementary file 1 — Supplementary Material 1. [file 12889_2025_22447_MOESM1_ESM.docx]

Supplementary File: template for intervention description and replication (TIDieR) checklist and guide, as described by Hoffman and colleagues(1)

| Item Number | Brief | Item | Action |
| --- | --- | --- | --- |
| 1 | Item Name | Provide the name or a phrase that describes the intervention | Lorn and Oban Healthy Options |
| 2 | Why | Describe any rationale, theory, or goal of the elements essential to the intervention | 12-week programme of exercise rehabilitation to support people with an unstable or unmanaged condition through extensive support and encouragement to enable people to be more physically active. |
| 3 | What: Materials | Describe any physical or informational materials used in the intervention, including those provided to participants or used in intervention delivery or in training of intervention providers. | Physical materials:   - Gym space - Public spaces for walking - Internet access   Informational materials:   - Written patient information leaflets - Powerpoint presentations to facilitate education element. |
| 4 | What: Procedures | Describe each of the procedures, activities, and/or processes used in the intervention, including any enabling or support activities | The programme includes an initial 1:1 consultation (with a health and wellbeing practitioner) to collect baseline data (see figure 1) and to tailor the core programme to meet individual needs. The programme includes twice weekly group-based (or online) exercise classes (including strength gym based classes, Tai Chi, walking and swimming) and a once weekly educational workshops across a range of health and wellbeing topics (that integrate behaviour change techniques). |
| 5 | Who Provided | For each category of intervention provider (such as psychologist, nursing assistant), describe their expertise, background, and any specific training given | The intervention is delivered by a team of exercise specialists (n=5). They have achieved level 3 or 4 CISMPA approved qualifications. |
| 6 | How | Describe the modes of delivery (such as face to face or by some other mechanism, such as internet or telephone) of the intervention and whether it was provided individually or in a group | During the intervention period classes were delivered using a combination of face-to-face and online formats, seeking to address equality determinants and accounting for disruption of the COVID-19 pandemic. For individuals who could not attend face-to-face sessions (for example due to health, geography or caring responsibilities), home-based exercise programmes were provided. |
| 7 | Where | Describe the type(s) of location(s) where the intervention occurred, including any necessary infrastructure or relevant features | The online element could be accessed by the individual anywhere with internet access.  The face-to-face element was delivered in a central location (Oban), at the local leisure centre. |
| 8 | When and How Much | Describe the number of times the intervention was delivered and over what period of time including the number of sessions, their schedule, and their duration, intensity, or dose | See Figure 1.  The intervention was a 12 week intervention with:   - 2x weekly physical activity training lasting roughly 60 minutes. These included online home-workouts, Tai Chi, gym-based classes, swimming and guided walks - 1x weekly educational sessions. |
| 9 | Tailoring | If the intervention was planned to be personalised, titrated or adapted, then describe what, why, when, and how | The programme includes an initial 1:1 consultation (with a health and wellbeing practitioner) to collect baseline data (see figure 1) and to tailor the core programme to meet individual needs. Participants were encouraged to attend as many of the three weekly events that they could, but there was no mandated or minimum attendance. Care was also taken to account for individuals’ health, economic and social circumstances during the initial 1:1 consultation. |
| 10 | Modifications | If the intervention was modified during the course of the study, describe the changes (what, why, when, and how) | The programme was designed to be inherently flexible, designed to meet need as determined at the initial 1:1 consultation.  The programme was affected by COVID-19 but continued, with delivery being provided online, during the lockdowns for 12 months between March 2020 and March 2021. |
| 11 | How Well: Planned | If intervention adherence or fidelity was assessed, describe how and by whom, and if any strategies were used to maintain or improve fidelity, describe them | Initial and review 1:1 consultations conducted by health and wellbeing practitioners allowed for a basic assessment of adherence and fidelity. During these consultations, baseline data were collected and the programme was tailored to individual participant needs. Attendance was monitored throughout the programme. Fidelity was maintained through an individualised approach, highly qualified staff ensure a high standard of delivery and adaptability to accommodate participants needs. |
| 12 | How Well: Actual | Actual: If intervention adherence or fidelity was assessed, describe the extent to which the intervention was delivered as planned | The intervention was impacted by COVID-19, resulting in a change in how the intervention was delivered. This had significant impacts on intervention adherence.  Furthermore, the intervention was designed to be flexible to meet individual needs, impacting the ability to clearly assess adherence. |

1. Hoffmann TC, Glasziou PP, Boutron I, Milne R, Perera R, Moher D, et al. Better reporting of interventions: template for intervention description and replication (TIDieR) checklist and guide. Bmj. 2014;348.
